# Supplementary material for: ZBTB38 requires an extended N-terminal zinc finger network to read mCpG- and discriminate TpG-containing DNA sequences
Source: J Biol Chem. 2026 Aug 28;302(10):113513. doi: 10.1016/j.jbc.2026.113513 (PMC13652841; doi:10.1016/j.jbc.2026.113513)
Supplement: Supplementary file 1 [file mmc1.pdf]

Supplemental material for

ZBTB38 requires an extended N-terminal zinc finger network to read mCpG- and discriminate TpG-containing DNA sequences

Jared Boster, Cooper Gangi, Nicholas O. Hudson, Dallin E. Billings,[1] Brandon Leonel Guerra Castañaza Jenkins, Victoria L. Ding, Bethany A. Buck[2]

From the Department of Chemistry, University of Utah, Salt Lake City, Utah 84112

[1]Current address: Department of Oncological Sciences, Huntsman Cancer Institute, University of Utah School of Medicine, Salt Lake City, UT, USA

[2]To whom correspondence should be addressed: Phone: 801-581-3186; Fax: 801-581-8433; E-mail: bethany.buck@utah.edu

Contains supplemental Tables S1 and supplemental Figures S1-S5.

Table S1. EMSA-SELEX sequencing results for the five N-terminal ZBTB38 zinc fingers with the methylated DNA pool*

mNZ38_1	GTCCAAGCTTCTGGAGGGTGAGTCAAAGGTAAGCTAAT**TGCGATA**ACTGACTTCACCCATGGCTAG
mNZ38_2	GTCCAAGCTTCTGGAGGGTGAGTGGCGTACTCA**TGACGCA**TCATAACTGACTTCACCCATGGCTAG
mNZ38_3	GTCCAAGCTTCTGGAGGGTGAGTATCCCTCCTCC**ACCCGCC**GATAACTGACTTCACCCATGGCTAG
mNZ38_4	GTCCAAGCTTCTGGAGGGTGAGTGGATAATTC**TTCCGCA**CGAATAACTGACTTCACCCATGGCTAG
mNZ38_5	GTCCAAGCTTCTGGAGGGTGAGTGGCACCACT**ATCCGCA**GCTATAACTGACTTCACCCATGGCTAG
mNZ38_6	GTCCAAGCTTCTGGAGGGTGAGTAAGAGCGAGGTT**TCACGCC**ATAACTGACTTCACCCATGGCTAG
mNZ38_7	GTCCAAGCTTCTGGAGGGTGAGTGGACCACAC**AGCGTCA**TCTATAACTGACTTCACCCATGGCTAG
mNZ38_8	GTCCAAGCTTCTGGAGGGTGAGTTCCGGGCACT**GGCGGCA**TAATAACTGACTTCACCCATGGCTAG
mNZ38_9	GTCCAAGCTTCTGGAGGGTGAGTACTCCATTAA**TCCCGCA**ACATAACTGACTTCACCCATGGCTAG
mNZ38_10	GTCCAAGCTTCTGGAGGGTGAGTATCATGTCCCA**TAGCGCC**AATAACTGACTTCACCCATGGCTAG
mNZ38_11	GTCCAAGCTTCTGGAGGGTGAGTGCAACG**CGCGGTT**CCATACATAACTGACTTCACCCATGGCTAG
mNZ38_12	GTCCAAGCTTCTGGAGGGTGAGTGGATCAGCACT**TTCCGCT**CATAACTGACTTCACCCATGGCTAG
mNZ38_13	GTCCAAGCTTCTGGAGGGTGAGTT**AGCGGTA**ACGCTGCCTCGATAACTGACTTCACCCATGGCTAG
mNZ38_14	GTCCAAGCTTCTGGAGGGTG**AGTCGCC**TAGCTCTCGAGTGTCATAACTGACTTCACCCATGGCTAG
mNZ38_15	GTCCAAGCTTCTGGAGGGTGAGTAC**TGACGCT**ATGCCCGCCAATAACTGACTTCACCCATGGCTAG
mNZ38_16	GTCCAAGCTTCTGGAGGGTGAGTAAACCCAGCA**TCCCGCG**CCATAACTGACTTCACCCATGGCTAG
mNZ38_17	GTCCAAGCTTCTGGAGGGTGAGTCTGCTCCCCTAC**ACCCGCC**ATAACTGACTTCACCCATGGCTAG
mNZ38_18	GTCCAAGCTTCTGGAGGGTGAGTTCCCT**CACCGCG**AGGTGGTATAACTGACTTCACCCATGGCTAG
mNZ38_19	GTCCAAGCTTCTGGAGGGTGAGTAACCAG**GCACGCC**GCGTTGATAACTGACTTCACCCATGGCTAG
mNZ38_20	GTCCAAGCTTCTGGAGGGTGAGTTGGCA**CAACGCC**ACCTGCTATAACTGACTTCACCCATGGCTAG
mNZ38_21	GTCCAAGCTTCTGGAGGGTGAGTAGC**ATCCGCC**CATTTCCTAATAACTGACTTCACCCATGGCTAG
mNZ38_22	GTCCAAGCTTCTGGAGGGTGAGTACTGGGCCCAAG**ACCCGCC**ATAACTGACTTCACCCATGGCTAG
mNZ38_23	GTCCAAGCTTCTGGAGGGTGAGTGGT**AGCGTAT**GGCAACAGTATAACTGACTCCACCCATGGCTAG
mNZ38_24	GTCCAAGCTTCTGGAGGGTGAG**TATCGCT**TTAAAC**ACTCGCT**ATAACTGACTTCCCCCATGGCTAG
mNZ38_25	GTCCAAGCTTCTGGAGGGTGAGTAGCCTATC**ACCCGCA**GCCAATAACTGACTTCACCCATGGCTAG
mNZ38_26	GTCCAAGCTTCTGGAGGGTGAGTC**ATACGCC**CGACGTCAGGAATAACTGACTTCACCCATGGCTAG
mNZ38_27	GTCCAAGCTTCTGGAGGGTGAGTCGGCA**GGCGGAT**TCCCATTATAACTGACTTCACCCATGGCTAG
mNZ38_28	GTCCAAGCTTCTGGAGGGKGAGTTG**TGCCGCA**CTATGTCTCCATAACTGACTTCACCCATGGCTAG
mNZ38_29	GTCCAAGCTTCTGGAGGGTGAGTACTAGT**AGCCGCT**GCCGTTATAACTGACTTCACCCATGGCTAG
mNZ38_30	GTCCAAGCTTCTGGAGGGTGAGTCCAGTAAGGTT**GGCGGCA**CATAACTGACTTCACCCATGGCTAG
mNZ38_31	GTCCAAGCTTCTGGAGGGTGAGTATA**AGCCGCG**TTCCCTGCAATAACTGACTTCACCCATGGCTAG
mNZ38_32	GTCCAAGCTTCTGGAGGGTGAGTATCTAGAACTAAG**GACCGCA**TAACTGACTTCACCCATGGCTAG
mNZ38_33	GTCCAAGCTTCTGGAGGGTGAGTAGC**CATCGCA**ACGGTAAGCATAACTGACTTCACCCATGGCTAG
mNZ38_34	GTCCAAGCTTCCGGAGGGTGAGTCACCAC**CTTCGCT**CGCCACATAACTGACTTCACCCATGGCTAG
mNZ38_35	GTCCAAGCTTCTGGAGGGTGAGTACCAC**AGCGCGT**TGTGAGTATAACTGACTTCACCCATGGCTAG
mNZ38_36	GTCCAAGCTTCTGGAGGGTGAGTAAACG**TTCCGCC**CGTGACCATAACTGACTTCACCCATGGCTAG
mNZ38_37	GTCCAAGCTTCTGGAGGGTGAGTCCCCTA**CTCCGCT**AGTGGGATAACTGACTTCACCCATGGCTAG
mNZ38_38	GTCCAAGCTTCTGGAGGGTGAGTCGATGCAACGG**GGCGGCC**CATAACTGACTTCACCCATGGCTAG
mNZ38_39	GTCCAAGCTTCTGGAGGGTGAGTGTAA**TACCGCC**AACTCGATATAACTGACTTCACCCATGGCTAG
mNZ38_40	GTCCAAGCTTCTGGAGGGTGAGTTGCCCATACA**CACCGCA**CTATAACTGACTTCACCCATGGCTAG
mNZ38_41	GTCCAAGCTTCTGGAGGGTGAGT**TGACGCG**TACGGAATCCACATAACTGACTTCACCCATGGCTAG
mNZ38_42	GTCCAAGCTTCTGGAGGGTGAGTGGCGTCAACC**TACCGCA**CCATAACTGACTTCACCCATGGCTAG
mNZ38_43	GTCCAAGCTTCTGGAGGGTGAGT**GTACGCC**AATGACGTTGGCATAACTGACTTCACCCATGGCTAG
mNZ38_44	GTCCAAGCTTCTGGAGGGTGAGTCGCATA**TCCCGCC**GCTTGGATAACTGACTTCACCCATGGCTAG
mNZ38_45	GTCCAAGCTTCTGGAGGGTGAGTAGCCCGTAGCCT**TAGCGCC**ATAACTGACTTCACCCATGGCTAG
mNZ38_46	GTCCAAGCTTCTGGAGGGTGAG**TGCGCCA**CCAACATTCTGTTATAACTGACTTCACCCATGGCTAG
mNZ38_47	GTCCAAGCTTCTGGAGGGTGAGTTGCAGAAGAGAA**TAGCGCT**ATAACTGACTTCACCCATGGCTAG
mNZ38_48	GTCCAAGCTTCTGGAGGGTGAGTGCCTTTACCCTG**TTACGCC**ATAACTGACTTCACCCATGGCTAG

*Primer flanks are underlined in each sequence while the consensus sites are in bold.

A

KBS (ZBTB33): TCCTGCNA

Z4BS (ZBTB4): TCCTGCNA

E-box (ZBTB38): ACCTGCCT

B

```
   335
   AEVPPL VYNCSCCSKA FDSSTLLSAH MQLHKPTQEP LVCKYCNKQF TTLNRLDRHE QICMRSSHMP

403                                       441
IPGGNQRFLE NYPTIGQNGG SFTGPEPLLS ENRIGEFSST GSTLPDTDHM VKFVNGQMLY SCVVCKRSYV

TLSSLRRHAN VHSWRRTYPC HYCNKVFALA EYRTRHEIWH TGERRYQCIF CLETFMTYYI LKNHQKSFHA

                           570
IDHRLSISKK TANGGLKPSV YPYKLYRLLP
```

Figure S1. *A*, Sequence alignment of the confirmed (ZBTB33 (1-3) and ZBTB4 (4)) and proposed (ZBTB38 (5)) core TpG-containing DNA consensus motifs for the three ZBTB methyl-CpG binding protein family members. For ZBTB38, the depicted E-box core sequence is the one utilized in this study (Table 1, E-box R). *B*, Amino acid sequence of human ZBTB38 (UniProt ID: Q8NAP3) spanning the N-term ZF region utilized for protein construct design in this study. Zinc coordinating cytosine and histidine residues for the five N-term ZFs are represented in gold and magenta, respectively.

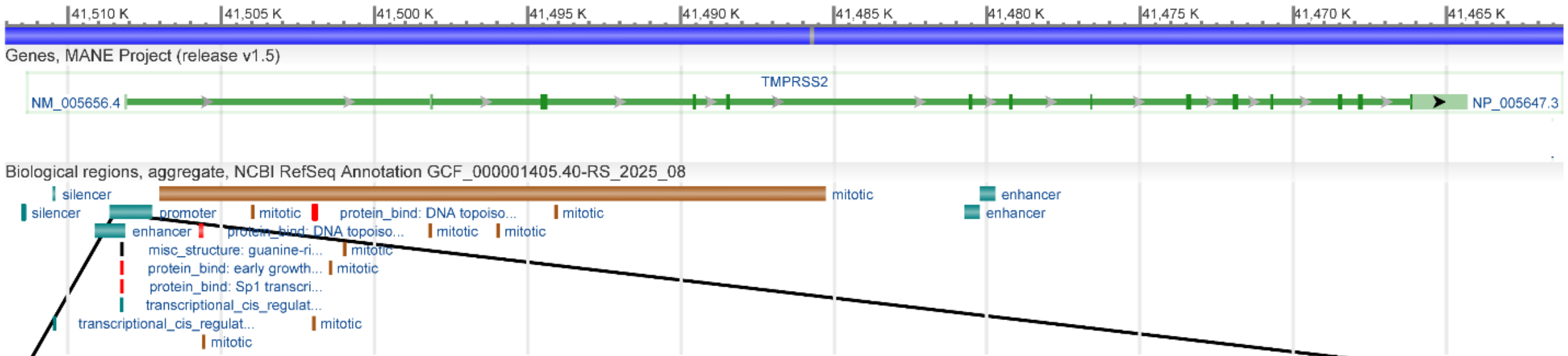

>NC_000021.9:41507255-41508648 Homo sapiens chromosome 21, GRCh38.p14 Primary Assembly

GCCGGGCAGGACAGGATGAGGTGGACCGAAGCGCCCAGGTGCCCCGCCTTCCCCCCAACCCGGCACGGAG
CGCAAGCGGAGGAGCCAGACCGGCTCCTCCCGGGCGGGTTCCTGCCGGTGCCGGGTGTCCAGGAGGCCCC
GGCCCTGATCGCCGCCAGCGGGTCGCCGCAACGGGGCCTGAGTCGCGAGCAGTCCCTCCCCGAGACTGAC
TGGCCGTTCGCACCTCGCCCGGGGGCCCTCCAGCAGCCTGGCCCAGCGGTCCCCGCCGCGCCGGCCGTAT
CGTTCCCGGCATCTCAGCGAGTTTCCAGTTTCTCCCTACCTCAGTAGGGAACCAAGAAGCAGAAGGAGTT
CAGGTAACACTGCGGGACTGCAGAGATTTCCCCCAAACAAAGAAGCCA**ACCGAC**AGCACTCCCAGTCCTC
CTCCCCAAAGAGAAAAGGCGCACCGGTGCTCCCAGGCGGGGGCCGTGGAGGGCAGGCGGACTAGGAGCCA
GCTTTGGGGACCCCGGGGGACTCTCTTCCACCAACTGGCTAGGGACTCGCAGGACCACCTGCCCCAACCT
CGCGATGCCAAGCCAACAGGGGCGGCGAGGGCTCTCGGCCGTGCGCAAGGGGTCCCAGGCGCCCAGCACT
CTCCCAGCACCCCGGGAGGCGCCCTGCCCGGCTGGCCCCAGCGCTCGACCCTCGGGCGCACTCACCTGCC
GCGCCGCGCTCCTCACACCCGCTTTCACCTCCGGGCGGGGCAGGGGGCATCGGCGGGTCCCAGGCGCCCA
GGTTCCCCTCCCCAGCCCGGACCCCGAGCCGGGACCCTGGTACCGGCGCCGCTCACCTGCCGCGCTCCAG
GCGGCGCTCCCCGCCCCTCGCCCTCCGCCTCCGCCTCCGCCTCCTGCTTAGCTCGCGCCTACTCGGCCCG
GCCCGCCCTGGCTCTCGCTTTAATCTCAGGA**GGCGGTG**TCCCAGGGTCCAGGGCGACACCCCCAAGCGGG
TCGCGGGTCACCCTGGGCGGGCGGGGGCGGGGGCGGCGGGAGGAGGCGTTTCCCAAGGGAAGTCCGGGAC
GGGTTCCCTGCCTGGCTCAACCCCGGCTGCGGAGGGACCCATAAGGGACTCTGGGGCGCTTCCCGGGCCC
GGCCACAGCGGCCAACCTGCCCCTCTCCGGTCCGCGGCGCAGCGCTGCCCGAGGGCTCGAGTTTGGGTTA
AGGAAGGGGATTCTGGGGAGCGCTCCTGGAGTCCTGGGTGAGCCCCCGGAGCTGCTACCTCACGAGCTCC
TGTAGGCGGCCGGCCCCGTCTTGGCCTCCGGGGAACCCACTGTAGCCTCTGGGCAAGGACCCAGGGTAGG
CCTTGGGGACTGCTGGCCCAAGCACGAGTGGCGGGGACACAGCGCCGGACGCCCAAGACCCTCC

Figure S2. Gene annotation for the human *TMPRSS2* gene from the current GRCh38.p14 primary assembly (Ch 21; 41,508,158 – 41,464,305). The *TMPRSS2* promoter FASTA sequence (41,507,255 – 41,508,648) (6) is depicted below, denoting the locations of the ZBTB38 N- (*blue highlight*) and C-term (*green highlight*) ZF domain consensus sequences. The underlined region was used to design the mTMPRSS2 DNA target utilized in this study (Table 1). The top figure was generated using the NCBI Genome Data Viewer (reference sequence NC_000021.9) (7).

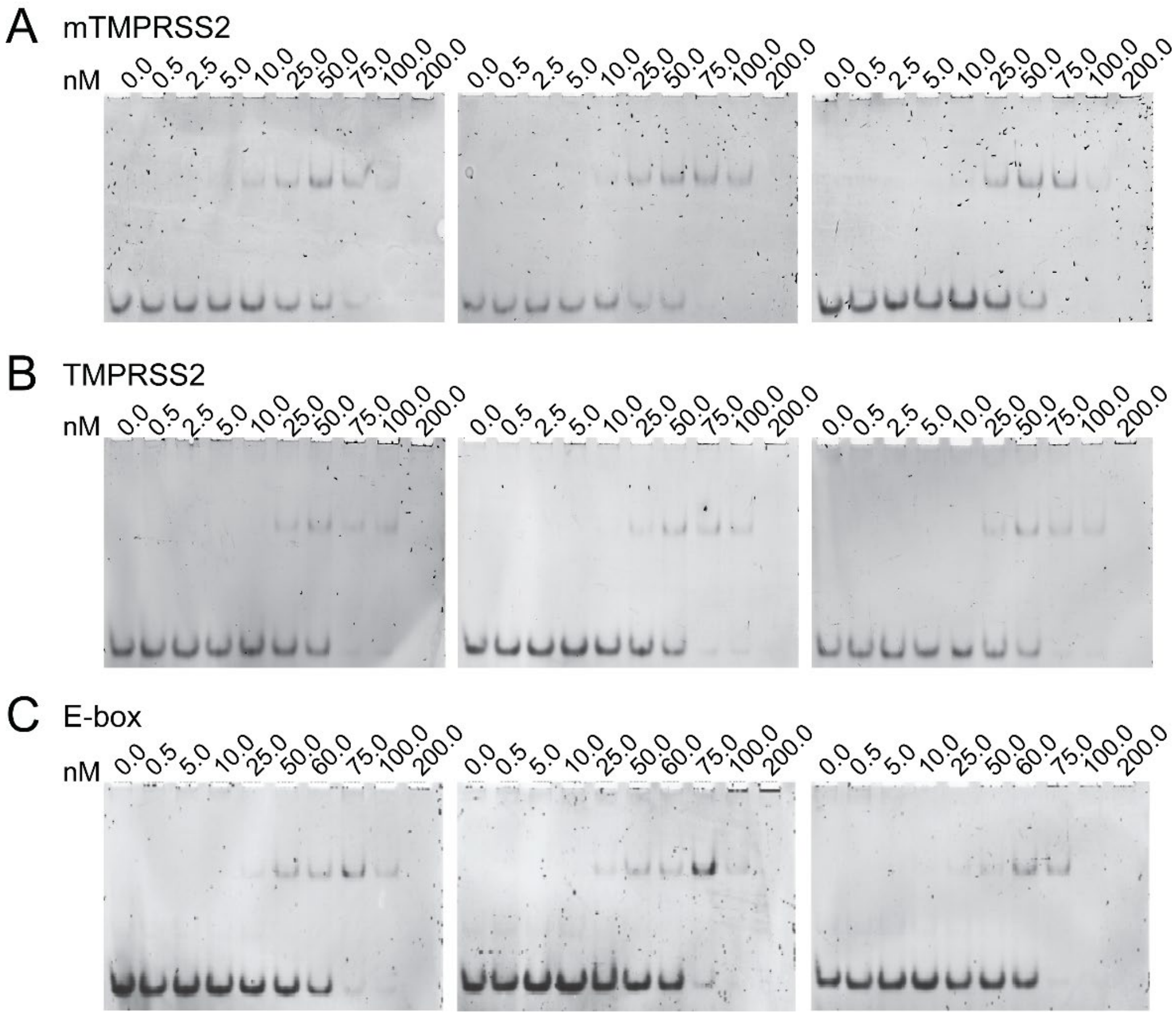

Figure S3. EMSA gel triplicates for ZBTB38 ZFs 1-5 binding to *A*, mTMPRSS2 (mCpG); *B*, TMPRSS2 (CpG); and *C*, E-box (TpG). These gels were used to generate binding isotherms and determine K_d values associated with Fig. 2*C*.

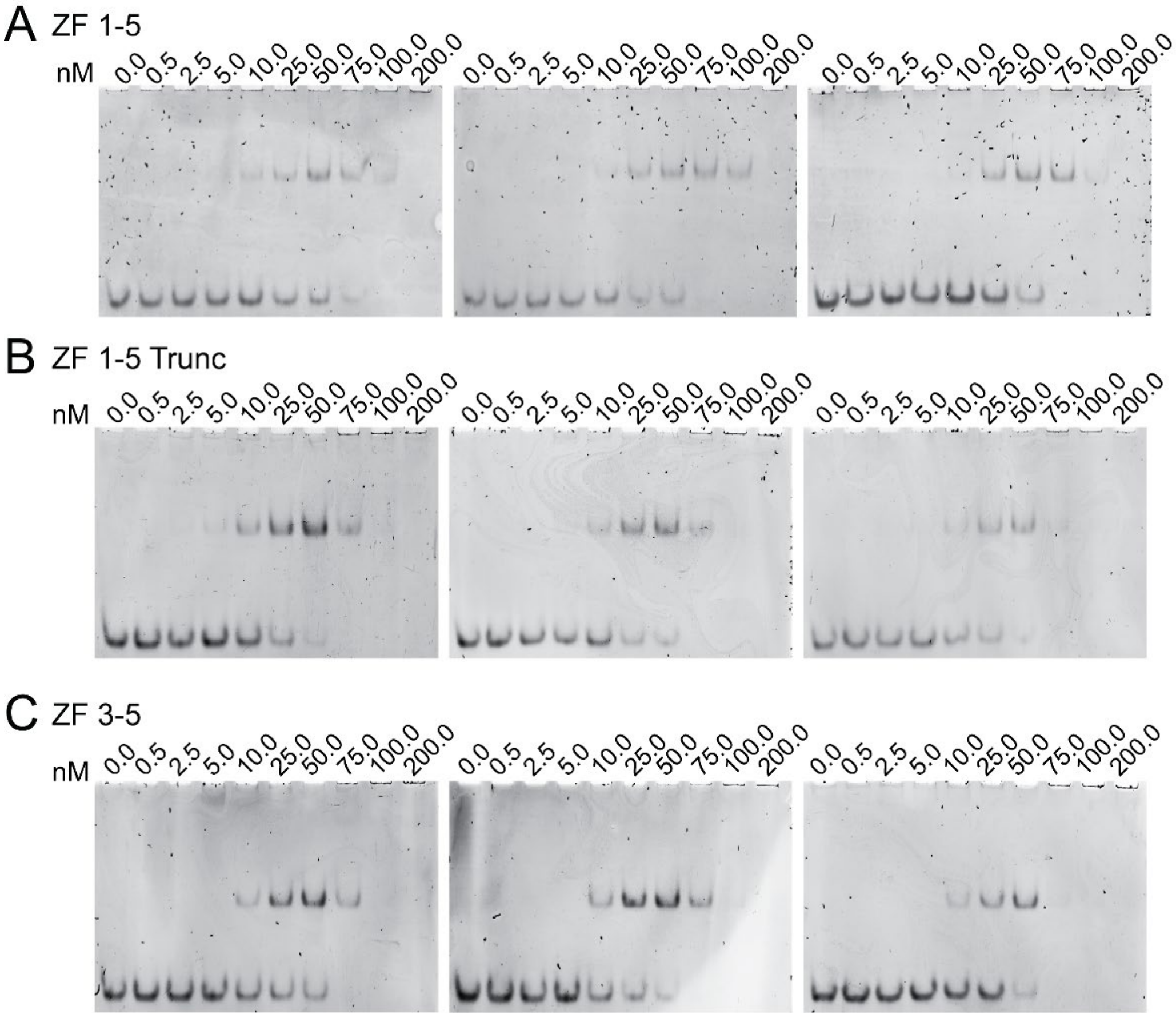

Figure S4. EMSA gel triplicates for ZBTB38 *A,* ZF 1-5; *B*, ZF 1-5 Trunc; and *C*, ZF 3-5 binding interactions with mTMPRSS2. The gels in *A* are reproduced from Fig. S2*A* for comparison purposes. These gels were used to generate binding isotherms and determine K_d values associated with Fig. 3*D*.

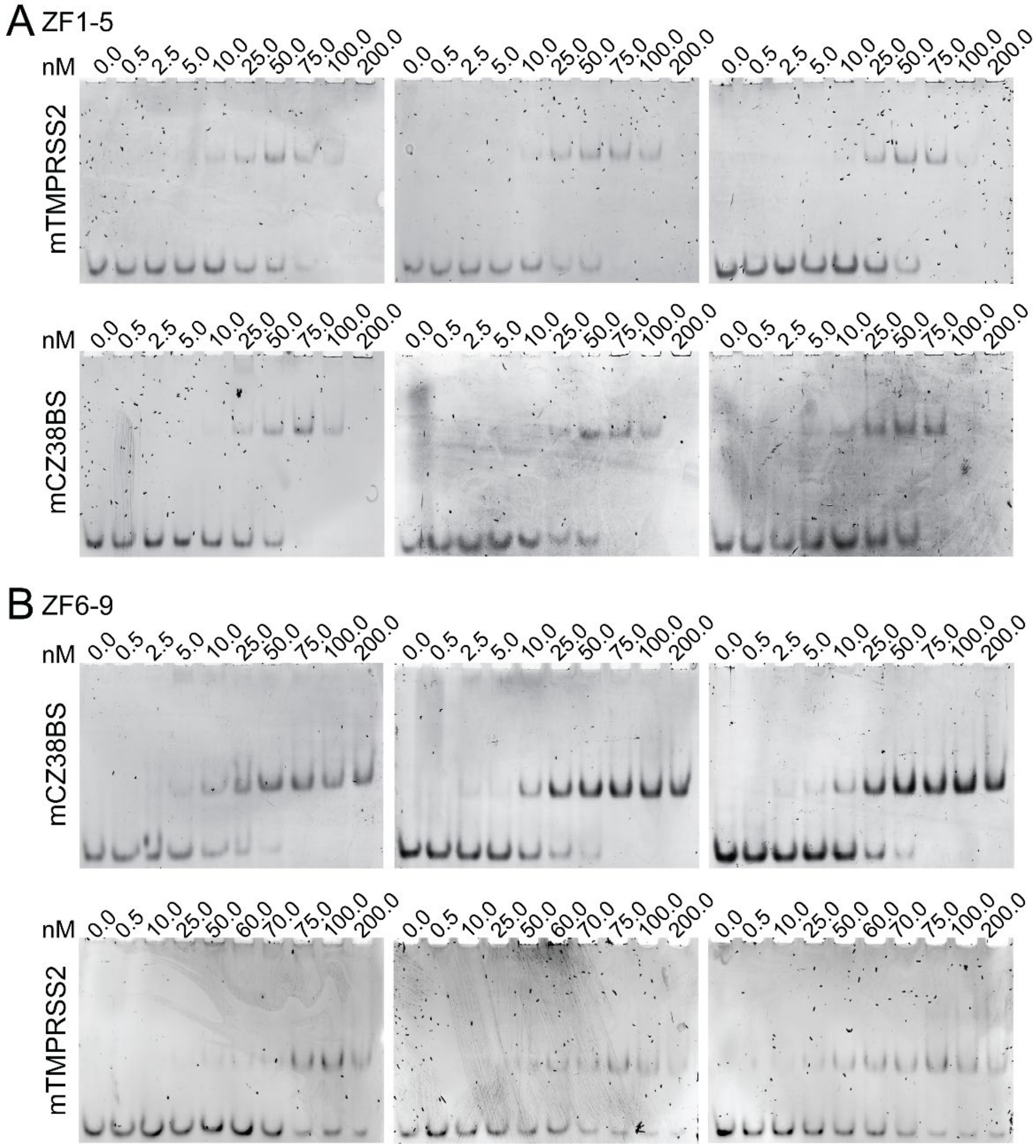

Figure S5. EMSA gel triplicates for *A*, ZBTB38 ZF 1-5 binding to mTMPRSS2 (*top*) or the ZBTB38 C-term ZF consensus sequence (mCZ38BS (8); *bottom*) and *B*, ZBTB38 ZFs 6-9 binding to mCZ38BS (*top*) or mTMPRSS2 (*bottom*). The ZF 1-5:TMPRSS2 gels in *A* are reproduced from Fig. S2*A* for comparison purposes. These gels were used to generate binding isotherms and determine K_d values associated with Fig. 4.

References

1. Buck-Koehntop, B. A., Martinez-Yamout, M. A., Dyson, H. J., and Wright, P. E. (2012) Kaiso uses all three zinc fingers and adjacent sequene motifs for high affinity binding to sequence-specific and methyl-CpG DNA targets *FEBS Lett* **586**, 734–739.
2. Buck-Koehntop, B. A., Stanfield, R. L., Ekiert, D. C., Martinez-Yamout, M. A., Dyson, H. J., Wilson, I. A. *et al.* (2012) Molecular Basis for recognition of methylated and specific DNA sequences by the zinc finger protein Kaiso *Proc Natl Acad Sci USA* **109**, 15229–15234.
3. Daniel, J. M., Spring, C. M., Crawford, H. C., Reynolds, A. B., and Baig, A. (2002) The p120^{ctn}-binding partner Kaiso is a bi-modal DNA-binding protein that recognizes both a sequence-specific consensus and methylated CpG dinucleotides *Nucleic Acids Res* **30**, 2911–2919.
4. Sasai, N., Nakao, M., and Defossez, P.-A. (2010) Sequence-specific recognition of methylated DNA by human zinc-finger proteins *Nucleic Acids Res* **38**, 5015–5022.
5. Kiefer, H., Chatail-Hermitte, F., Ravassard, P., Bayard, E., Brunet, I., and Mallet, J. (2005) ZENON, a novel POZ Kruppel-like DNA binding protein associated with differentiation and/or survivial of late postmitotic neurons *Mol Cell Biol* **25**, 1713–1729.
6. Shen, L. W., Qian, M. Q., Yu, K., Narva, S., Yu, F., Wu, Y. L. *et al.* (2020) Inhibition of Influenza A virus propagation by benzoselenoxanthenes stabilizing TMPRSS2 Gene G-quadruplex and hence down-regulating TMPRSS2 expression *Sci Rep* **10**, 7635.
7. Sayers, E. W., Beck, J., Bolton, E. E., Brister, J. R., Chan, J., Connor, R. *et al.* (2025) Database resources of the National Center for Biotechnology Information in 2025 *Nucleic Acids Res* **53**, D20–D29.
8. Pozner, A., Hudson, N. O., Trewhella, J., Terooatea, T. W., Miller, S. A., and Buck-Koehntop, B. A. (2018) The C-terminal zinc fingers of ZBTB38 are novel selective readers of DNA methylation *J Mol Biol* **430**, 258–271.
